# Inhibition of Tyrosinase by Mercury Chloride: Spectroscopic and Docking Studies

**DOI:** 10.3389/fphar.2020.00081

**Published:** 2020-03-06

**Authors:** Jianmin Chen, Yaling Ye, Mengnan Ran, Qinglian Li, Zhipeng Ruan, Nan Jin

**Affiliations:** ^1^ School of Pharmacy and Medical Technology, Putian University, Fujian, China; ^2^ Key Laboratory of Pharmaceutical Analysis and Laboratory Medicine, Putian University, Fujian Province University, Fujian, China

**Keywords:** mercury chloride, tyrosinase, spectroscopic measurements, molecular docking, inhibition mechanism

## Abstract

Inorganic mercury compounds have been used in skin-lightening products since ancient times. Although a previous study demonstrated that mercury impeded the transfer of Cu^2+^ to the apotyrosinase, the effect of mercury on tyrosinase is still unclear. In the present study, the mechanism of mercury chloride (HgCl_2_) induced inactivation of tyrosinase was investigated for the first time. The IC_50_ values were 29.97 and 77.93 μmol/L for monophenolase and diphenolase, respectively. A kinetic analysis revealed that HgCl_2_ inhibited tyrosinase activity in an irreversible non-competitive manner. The strong intrinsic fluorescence quenching suggested that the formation of the HgCl_2_-tyrosinase complex induced conformational changes of the enzyme, and HgCl_2_ had only one single binding site or a single class of binding site on tyrosinase. The molecular docking and further experiments demonstrated that HgCl_2_ bound to the amino residuals (His) in the catalytic center of tyrosinase. To our knowledge, these findings presented in this paper were the first evidence of the direct interactions between HgCl_2_ and tyrosinase, which provided a deep understanding of the inhibition mechanism of mercury on tyrosinase.

## Introduction

Tyrosinase (EC 1.14.18.1), a type III copper protein, is ubiquitously distributed in bacteria, fungi, plants, insects, crustaceans, and mammals (Yoon et al., 2009). It is able to perform two successive reactions: the hydroxylation of tyrosine to dopa (monophenolase activity) and also the subsequent oxidation of dopa to dopaquinone (diphenolase activity) (Körner and Pawelek, 1982; Tan et al., 2016). Dopaquinone, also known as o-dopaquinone, is of high reactivity and its derivatives spontaneously polymerize to form melanin through a series of chemical reactions (Seo et al., 2003). Consequently, tyrosinase plays a pivotal role in melanin formation, which is an important component responsible for skin pigmentation in humans. Although melanin produced in skin melanocytes provides protection from UV radiation, excessive accumulation of melanin may cause cosmetic flaws such as age spots, freckles, senile lentigines, solar lentigo, and melasma in humans (Germanò et al., 2012; Hridya et al., 2016; Soares et al., 2017).

To decrease the abnormal accumulation of melanin, the use of skin-lightening (also known as skin bleaching and skin whitening) products has been prevalent among women all over the world for decades (Olumide et al., 2008). The active ingredients known to be effective for skin lightening include various corticosteroids, hydroquinone, and mercury (Barry et al., 2011). Mercury exists primarily in three forms: inorganic, organic, and metallic (or elemental) (Boyd et al., 2000). Inorganic mercury compounds, including mercuric chloride, mercurous chloride, and mercurous oxide, have been used in skin-lightening products since ancient times (Kibukamusoke et al., 1974). These compounds occur in a mercuric (Hg II) or mercurous (Hg I) form and have toxic effects on humans (Clarkson et al., 2003). It has been discovered that the use of skin-lightening products is one of the causes of mercury toxicity (Agrawal and Mazhar, 2015). More specifically, the use of skin-lightening products containing inorganic mercury may lead to central nervous system, gastrointestinal and renal toxicity (Chan, 2011). For this reason, the sale of skin-lightening products containing inorganic mercury was prohibited in many countries (Al-Saleh, 2016). Despite repeated warnings of mercury poisoning by health authorities and the ban against the sale of skin-lightening products containing inorganic mercury, these products are widely available for sale in pharmacies, beauty aid stores and on the internet (Hamann et al., 2014).

The failure in the prohibition of adding inorganic mercury to skin-lightening products is an interesting question worthy of consideration. We believe that the main reason may be the good performance of inorganic mercury compounds in skin-lightening and anti-freckle effects. So now comes the question, why do these compounds exhibit such strong inhibition ability of melanin production. It is well known that mercury can replace the copper needed for tyrosinase activity, thereby deactivating enzymes and producing skin-lightening effects (Lerner, 1952a). Specifically, mercury ions can compete with copper ions for active centers on apotyrosinase to produce an inactive preparation; it is worth noting that once copper becomes attached to the enzyme it is replaced by ion of mercury with difficulty (Lerner, 1952b). That is to say, the previous literature (Lerner, 1952b) has demonstrated that mercury only displayed an effect on apotyrosinase as opposed to tyrosinase. Therefore, the direct interaction between mercury and tyrosinase is still unknown. In this study, we used kinetics measurements, spectral investigation, and molecular docking to study the effects of mercury on tyrosinase and tried to elucidate the intrinsic mechanism of the interaction at the molecular level.

## Materials and Methods

### Materials

Tyrosine (98%), dopa (98%), and mushroom tyrosinase (1000U/mg, EC 1.14.18.1) were purchased from Sigma-Aldrich (St. Louis, MO, USA). Mercuric chloride (HgCl_2_, 99.5%) and kojic acid (99%) was obtained from Aladdin Bio-technology (Shanghai, China). Histidine (His), valine (Val), glutamate (Glu), and alanine (Ala) were of analytical grade and bought from Xiya Chemical Industry (Shandong, China). All other chemicals and reagents used were of analytical grade or pharmaceutical grade. A Milli-Q-Plus ultra-pure water system from Millipore (Sartorius 611, Germany) was used throughout the study to obtain water used during the experiments.

### The Effects on the Monophenolase Activity of Tyrosinase

The method was modified based on the previous description (Chen et al., 2016). In brief, the assays were carried out by using 3 mL of reaction system containing 0.5 mmol/L substrate (tyrosine) in 50 mmol/L Na_2_HPO_4_-NaH_2_PO_4_ buffer solution (PBS, pH = 6.8) at 305 K. A 100 μL aliquot of HgCl_2_ solutions with various concentrations (0, 1.25, 5 and 10 mmol/L) was added to the reaction system to give concentrations ranged from 0 to 333.33 μmol/L. The final concentration of tyrosinase was 33.33 U/mL. The optical densities (OD) at wavelength of 475 nm were recorded using UV spectrophotometer (UV 2550, SHIMADZU) every 30 s in the first 2 mins and then every 1 min until 30 mins. Kojic acid was used as a positive control. The appropriate blanks (containing both tyrosine and HgCl_2_ but without tyrosinase) were tested and subtracted to correct the absorbance.

### The Effects on the Diphenolase Activity of Tyrosinase

The method was performed as previously described (Chen et al., 2017). The assays were carried out by using 3 mL of reaction system containing 0.5 mmol/L substrate (dopa) in 50 mmol/L PBS (pH = 6.8) at 305 K. The final concentration of tyrosinase was 13.33 U/mL. A 100 μL aliquot of HgCl_2_ solutions with various concentrations (0.15, 0.31, 0.625, 1.25, 5, 10, and and 20mmol/L) was added to the reaction system to give concentrations ranged from 10.42 to 666.67 μmol/L. The OD values of the reaction system in the presence of HgCl_2_ solutions (A_1_) were recorded at the wavelength of 475 nm using the UV spectrophotometer. The OD values of the reaction system in the absence of HgCl_2_ and tyrosinase were used as a positive (A_2_) and a negative (A_3_) control, respectively. Relative enzymatic activity (%) = (A_1_-A_3_)/A_2_×100%. The experiment was repeated three times. The IC_50_ value was calculated as the concentration (leading to achieve 50% inhibition) according to the equation, which is obtained by curve fitting of the enzyme activity versus the concentrations of inhibitor.

### Immobilized Tyrosinase for Analysis of Inhibitory Mechanism

Experiments were conducted to confirm the inhibitory mechanism of HgCl_2_ on tyrosinase furtherly by using immobilized tyrosinase. In brief, we added 200 µL of diluted tyrosinase (2500 U/mL) to each well of a 24-well polystyrene plate and then covered the plate and incubated it at 4°C overnight to produce immobilized tyrosinase. The plate was brought to room temperature, and we thoroughly decanted the solution from wells and washed the wells two times with PBS to remove the unfixed enzyme. We added 3 mL of inhibitor solution to each well and incubated them at room temperature for 30 mins to assure the fully reaction between the inhibitor and the enzyme, discarding the liquid following by washing the wells two times with PBS to remove the excess inhibitor. Then, 3 mL of PBS was added to each well, and they stood for 10 mins to make the dissociation of inhibitor-tyrosinase complex. WE thoroughly decanted the solution from wells to remove the dissociated inhibitor (the procedure was repeated three times). Finally, 3 mL of substrate (dopa) was added to each well and react at room temperature for 30 mins to test the residual activity of tyrosinase. The tested samples included PBS (negative control), 95% ethanol (positive control of irreversible inhibitor), kojic acid (positive control of reversible inhibitor), and HgCl_2_, and each sample was tested three times. The concentration of Kojic acid and HgCl_2_ was both 666.67 μmol/L.

### Kinetic Analysis for Non-Competitive Inhibition

The non-competitive inhibition type of tyrosinase was assayed by a Lineweaver–Burk plot, which can be described as the following equation: 

(1) $$\frac{1}{\nu }=\frac{K_{m}}{{\text{V}}_{\max}}\left(1+\frac{\left[\text{I}\right]}{K_{i}}\right)\frac{1}{\left[\text{S}\right]}+\frac{1+\frac{\left[\text{I}\right]}{K_{i}}}{{\text{V}}_{\max}}$$

and a secondary plot is presented below

(2) $$\text{In}.=\frac{\left[\text{I}\right]}{{\text{K}}_{\text{i}}{\text{V}}_{\max}}+\frac{1}{{\text{V}}_{\max}}$$

where ν is velocity of the enzyme reaction calculated by a change in absorbance at the wavelength of 475 nm per minute in the absence and presence of HgCl_2_. *K*_m_ and *K*_i_ are the Michaelis–Menten constant and inhibition constant, respectively. The values of *K*_m_ can be calculated from the equation (1). [I] and [S] are the concentrations of inhibitor and substrate, respectively. In. is the intercept value of the Lineweaver–Burk curve. *K*_i_ should be calculated by the secondary plot of In. vs. [I].

### Intrinsic Fluorescence Quenching

The fluorescence emission spectra were obtained using Spectrofluorometer FS5 (Edinburgh Instruments, England) equipped with a 150 W xenon lamp and a thermostat bath. We added a 2.0 mL aliquot of tyrosinase solution (400 U/mL) to the quartz cuvette (1 cm path length) and gradually added 2 mmol/L solution of HgCl_2_ to give concentrations ranged from 0 to 400 μmol/L. After five-minute incubation periods, fluorescence in the range of 290–500 nm was measured while the excitation wavelength was fixed at 280 nm. All the fluorescence data collected from these experiments were corrected for absorption of excitation light and re-absorption of emitted light according to the following equation: (Wang et al., 2015)

(3) $$F_{c}=F_{m}e^{\left(A_{1}+A_{2}\right)/2}$$

where *F*_c_ and *F*_m_ represent the corrected and measured fluorescence, respectively. A_1_ and A_2_ are the absorbance of HgCl_2_ at excitation and emission wavelengths, respectively.

### Molecular Docking Study

Molecular docking study was performed by using AutoDock 4.2.6. The X-ray crystal structure of *Agaricus bisporus* tyrosinase (PDB ID: 2Y9W) and the 3D structure of HgCl_2_ were both retrieved from the RCSB Protein Data Bank (Ismaya et al., 2011). All input files were prepared using an AutoDockTools (ADT) 1.5.4 package, and a charge of +2 was assigned for copper ions. In order to carry out the docking simulation, a 60 Å × 60 Å × 60 Å -point grid with a spacing of 0.375 Å centered at 4.827, 28.489, 92.878 Å was defined, which fully enclosed the catalytic center of tyrosinase. The AutoGrid program was used to construct the grid maps for energy scoring. The three-dimensional location and orientation of the HgCl_2_ was explored using the Lamarckian genetic algorithm (LGA). The docking results generated 100 conformations of HgCl_2_, which were grouped into clusters by a root mean square (RMS) deviation tolerance of 2.0. The lowest energy (optimal conformation) and the largest number cluster (suboptimal conformation) were chosen for further analyses with the PyMOL molecular graphics system.

### The Interactions Between HgCl_2_ and Amino Acid

According to the results of docking study, we furtherly checked the interactions between HgCl_2_ and amino acid. We assumed that if HgCl_2_ indeed binds to the amino acid residues of the enzyme, such as His, Val, Glu, and Ala, premixing the amino acid with HgCl_2_ would affect the inhibitory effects. Thus, the monophenolase and diphenolase activity of tyrosinase both were determined by adding the mixture of HgCl_2_ and amino acid. The mixture was prepared just before use by adding an aliquot of 200 μL HgCl_2_ solution to the equal volume of amino acid solution, and which was allowed to react for 10 mins at room temperature. Furtherly, to prove the interaction between His and HgCl_2_, the UV-Vis spectra of His, HgCl_2_, and the mixture of both were measured. In brief, 5 and 10 mmol/L solutions of His, HgCl_2_ were prepared beforehand, and the mixture was produced by mixing the His and HgCl_2_ solutions (10 mmol/L) in the volume ratio of 1:1. The spectra of the samples at the wavelengths between 200 and 800 nm were scanned by using UV spectrophotometer (UV 2550, SHIMADZU).The experiment was repeated three times.

### Statistical Analysis

Each data point of the experimental results, including tyrosinase activity assay, kinetic analysis for non-competitive type inhibition, fluorescence quenching test, and interactions between amino acid and HgCl_2_, was repeated at least three times. The data are presented as the mean ± SD (standard deviation). The statistical significance was determined at the level of P-value (< 0.05) by one-way analysis of variance.

## Results and Discussion

### Effects of HgCl_2_ on the Monophenolase Activity of Tyrosinase

**Figure 1:**
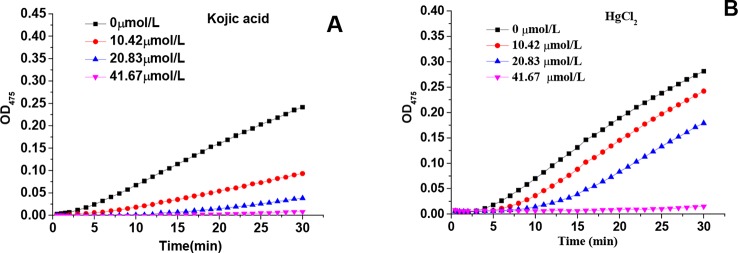
Effects of various concentrations of kojic acid and HgCl_2_ on monophenolase: progression of tyrosine oxidation by tyrosinase in the presence of various concentrations of kojic acid **(A)** and HgCl_2_ **(B)**.

**Figures 1A, B** showed the kinetic progression of tyrosine oxidation by tyrosinase in the presence of various concentrations of kojic acid and HgCl_2_, respectively. With the increase of kojic acid concentration, the lag time was prolonged from 1.44 min to approximately 10.33 min. Similarly, HgCl_2_ also exhibited a marked inhibitory effect with significant prolongation of the lag period from 2.00 to 13.4 mins. The inhibitory effects of HgCl_2_ on monophenolase were activity dependent on the concentrations because the steady rate (the slope of linear part of the kinetic equation) decreased with the increasing concentration of the inhibitors (Park et al., 2005). The IC_50_ values of kojic acid and HgCl_2_ were 13.10 and 29.97μmol/L, respectively, suggesting that HgCl_2_ had less inhibitory capability than kojic acid on monophenolase activity.

### Effect of HgCl_2_ on the Diphenolase Activity of Tyrosinase

**Figure 2:**
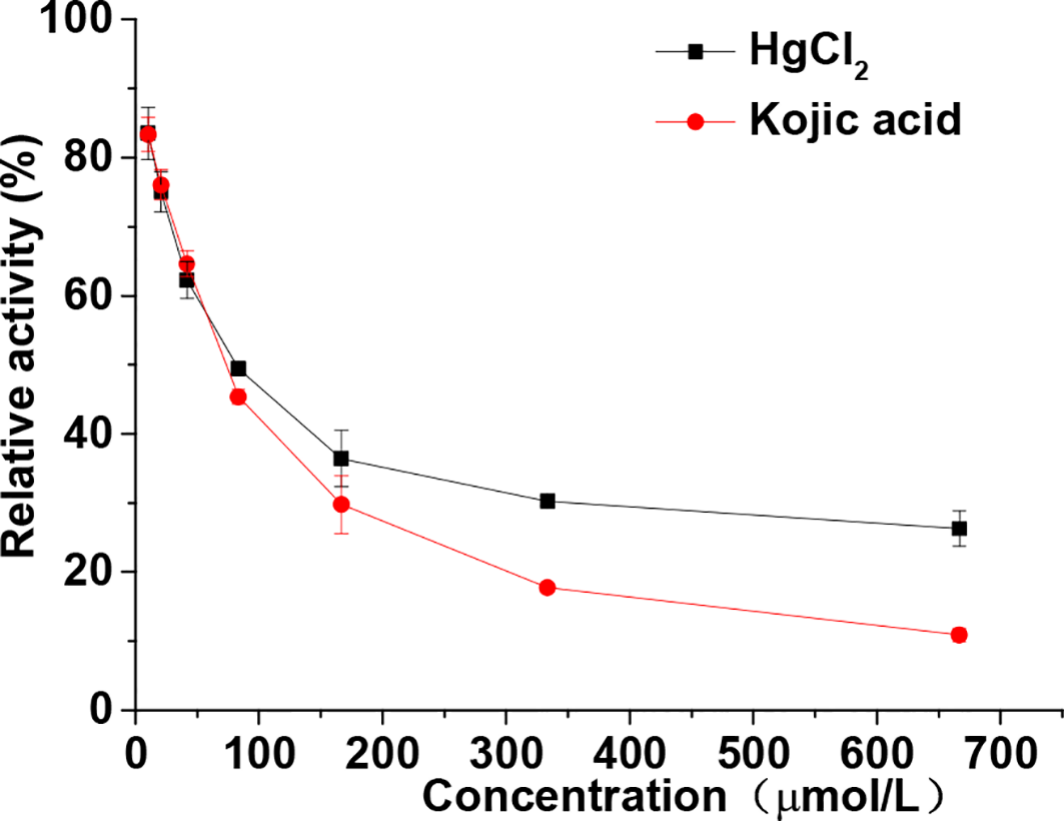
Relative activity of tyrosinase through diphenolase reactions with dopa in the presence of various concentrations (from 10.42–666.67 μmol/L) of HgCl_2_ and kojic acid. The final concentrations of dopa were 0.5 mmol/L. Data are presented as mean ± standard deviation (n = 3).

Both kojic acid and HgCl_2_ significantly inhibited the diphenolase activity in a dose-dependent manner, as shown in **Figure 2**. With an increase in the concentration of kojic acid and HgCl_2_, the diphenolase activities decreased rapidly with IC_50_ values of 73.05 and 77.93 μmol/L, respectively. HgCl_2_ exhibited significant inhibitory effect on the monophenolase activity also showed the same inhibitory effect on diphenolase activity, indicating it could inhibit the melanin synthesis in the early enzymatic stages. It is well-known that mercury replaces the copper required for tyrosinase activity, but once copper becomes attached to the enzyme it is replaced by ions of mercury with difficulty (Lerner, 1952b). That is to say, mercury was commonly considered to inhibit the melanin production by preventing the formation of the activated tyrosinase *via* replacement of copper ions. However, in this study, we found that mercury could inhibit both the monophenolase and diphenolase activity of tyrosinase by binding to the enzyme directly instead of replacing the copper ions required for tyrosinase.

### Inhibition Mechanism of HgCl_2_ on Tyrosinase

**Figure 3:**
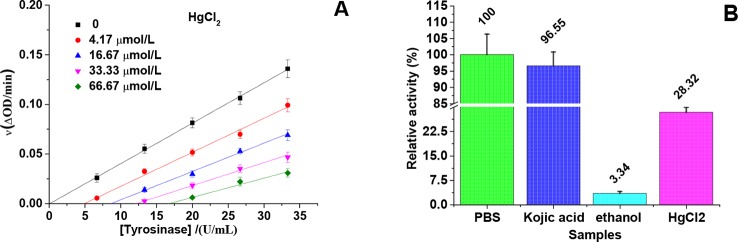
Inhibition mechanism of HgCl_2_ on tyrosinase. **(A)** An irreversible inhibition of the enzyme on a kinetic basis: the concentrations of HgCl_2_ were 0, 4.17, 16.67, 33.33, and 66.67 μmol/L for curves, and the concentrations of tyrosinase were 6.67, 13.33, 20, 26.67, and 33.33 U/mL. **(B)** Immobilized tyrosinase for analysis of inhibitory mechanism: As a negative control, the activity of tyrosinase with PBS treatment was defined as 100%, and the concentration of kojic acid and HgCl_2_ were both 666.67 μmol/L. The experiment was repeated three times (n = 3).

The relationship between enzyme activity (expressed as the rate of the oxidation reaction) and its concentration (6.67, 13.33, 20, 26.67, and 33.33 U/mL) in the presence of different concentrations of HgCl_2_ (0, 4.17, 16.67, 33.33, and 66.67 μmol/L) was investigated. A series of parallel (or roughly parallel) lines with the same slope and different abscissa intercepts (**Figure 3A**) were observed from the plots of tyrosinase activity versus the concentrations of enzyme in the presence of various concentrations of HgCl_2_, suggesting the inhibition of HgCl_2_ on tyrosinase was irreversible (Qiu et al., 2005). The increase of abscissa intercept indicated that the number of effective enzyme molecules decreased due to the irreversible inhibition.

In order to prove the irreversible inhibition furtherly, immobilized tyrosinase was used to study the effects of HgCl_2_ on tyrosinase, the results of which were shown in **Figure 3B**. As a negative control, the activity of tyrosinase treated by PBS was defined as 100%. The activity of tyrosinase treated by kojic acid returned to 96.55% because of the reversible inhibition, whilst treated by 95% ethanol only returned to 3.34% because of the irreversible inhibition. The activity of tyrosinase treated by HgCl_2_ returned to 28.32%, indicating HgCl_2_ indeed inhibits the tyrosinase in an irreversible way compared to kojic acid. However, the enzyme activity is much higher than the one treated by 95% ethanol, which may be due to the fact that the amount of the inhibitor (HgCl_2_) is not enough to inactivate the whole enzyme molecules coated on the plate.

### Inhibition Type of HgCl_2_ on the Tyrosinase

**Figure 4:**
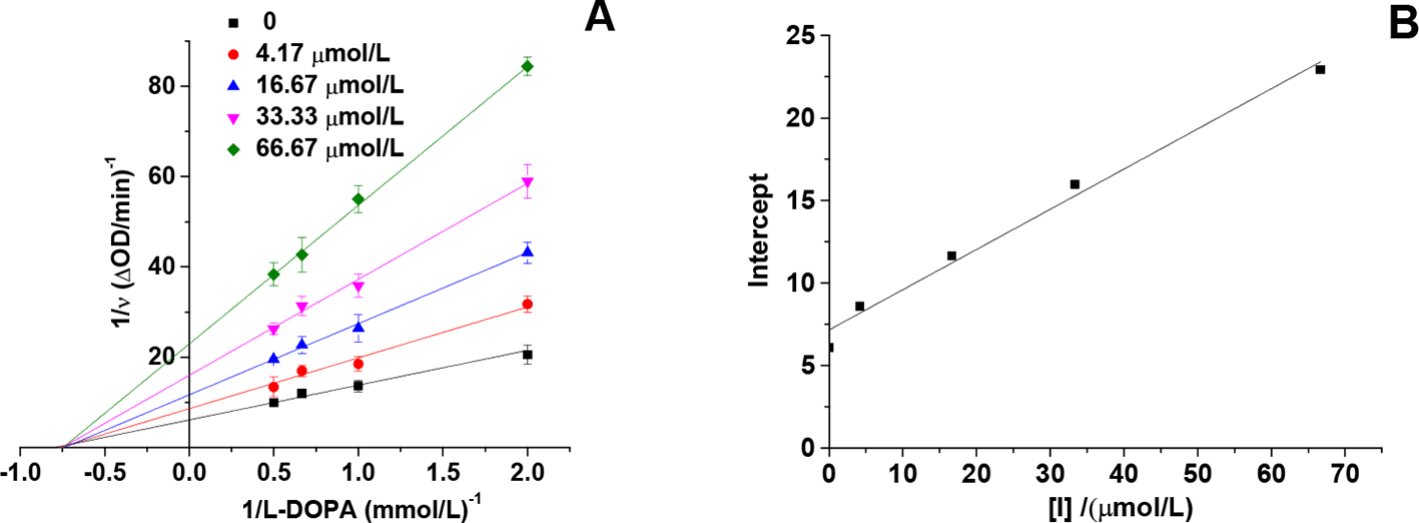
Lineweaver-Burk plot of mushroom tyrosinase in the presence of HgCl_2_: c(tyrosinase) = 13.33 U/mL, c(HgCl_2_) = 0, 4.17, 16.67, 33.33, and 66.67 μmol/L for curves, respectively. The experiment was repeated three times (n = 3).

The inhibitory effect of HgCl_2_ on the diphenolase activity was studied by the Lineweaver–Burk double reciprocal plots. As shown in **Figure 4A**, the horizontal axis intercept (-1/*K*_m_) keeps the value unchanged and the vertical axis intercept ($\frac{1+\frac{\left[\text{I}\right]}{K_{i}}}{{\text{V}}_{\max}}$) increases with the increasing inhibitor concentration, indicating that HgCl_2_ induced a non-competitive inhibition (Chen et al., 2005). Therefore, HgCl_2_ could bind both free enzyme and the enzyme-substrate complex with the same inhibition constants (*K*_i_). The value of *K*_i_ (29.41 μmol/L) was obtained from a plot of the vertical axis intercept (intercept of Lineweaver–Burk double reciprocal plots) versus the inhibitor concentration (Mu et al., 2013), as shown in **Figure 4B**.

### Effects of HgCl_2_ on Tyrosinase Conformational Change

**Figure 5:**
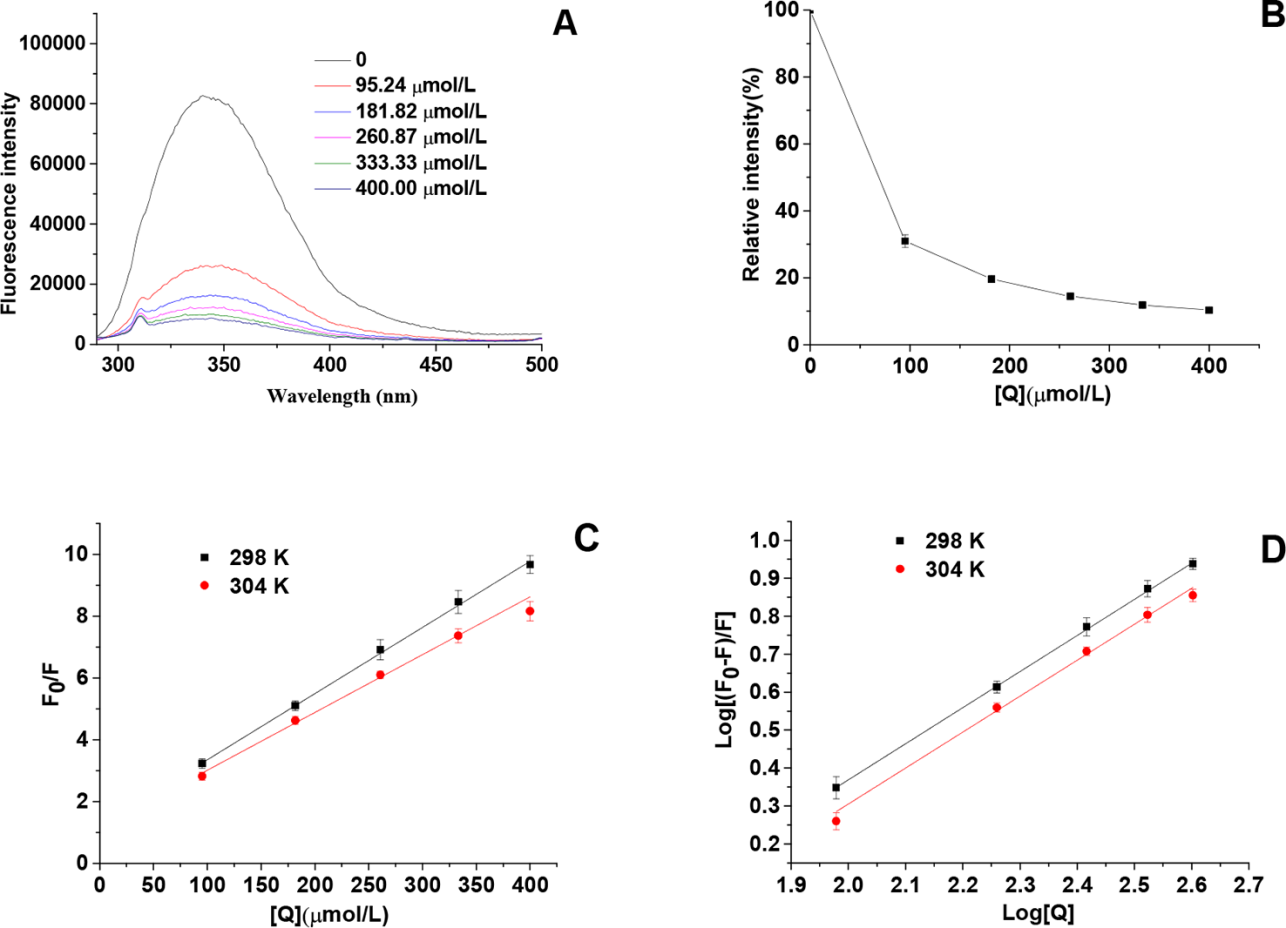
**(A)** Intrinsic fluorescence spectra of tyrosinase in the presence of HgCl_2_ at various concentrations, which were 0, 95.24, 181.82, 260.87, 333.33, and 400.00 μmol/L for curves, respectively. **(B)** Relative intensity (%) of tyrosinase at various concentrations of HgCl_2_ (298K). **(C)** The Stern–Volmer plots for the fluorescence quenching of tyrosinase at various temperatures (298 and 304 K). **(D)** Plots of log [(F_0_−F)/F] against log [Q] at various temperatures (298 and 304 K). F_0_ and F are the fluorescence intensities of tyrosinase in the absence and presence of HgCl_2_, and [Q] represents the concentration of HgCl_2_. The experiment was repeated three times (n = 3).

From the result of the intrinsic fluorescence spectra, we observed that HgCl_2_ bound to the tyrosinase resulted in conformational changes of tyrosinase: the spectra were gradually decreased in a dose-dependent manner (**Figures 5A, B**). The concentrations of HgCl_2_ for the curves (a–f) were 0, 95.24, 181.82, 260.87, 333.33, and 400.00 μmol/L, respectively. Although the decrease in the fluorescence intensity were caused by quenching, there were no significant wavelength shift with the accumulation of HgCl_2_, indicating that tyrosinase does not undergo conspicuous overall structural changes. Moreover, the fluorescence intensity decreased with the increasing concentration of the inhibitor because of the tryptophan-masking properties of HgCl_2_, which implied that the tyrosinase became disagglomerated, and its structure was loosened (Donghyun et al., 2006).

To identify the interaction mechanism of HgCl_2_ on tyrosinase, the data of fluorescence quenching were presented in the form of a Stern-Volmer plot, using the following equation:

(4) $$F_{0}/F=1+K_{\text{q}}{\text{τ}}_{0}\left[\text{Q}\right]=1+K_{\text{sv}}\left[\text{Q}\right]$$

where, *F*_0_ and *F* represent the steady-state fluorescence intensities in the absence and presence of HgCl_2_ (quencher), respectively, [Q] denotes the concentration of HgCl_2_, *K*_q_ is the quenching rate constant of the biomolecule (*K*_q_ = *K*_sv_/τ_0_), *K*_sv_ is the Stern–Volmer dynamic quenching constant, and τ_0_ is the average lifetime of the fluorophore in the absence of quencher (τ_0_ = 10^-8^ s). The value of *K*_sv_ determined by the linear regression plot of F_0_/F vs. [Q] at 298 and 304 K were (2.14 ± 0.05) × 10^4^ and 1.83 ± 0.03 × 10^4^ L/mol, respectively. The plot (**Figure 5C**) showed a good linear relationship, indicating that a single type of quenching process (static or dynamic quenching) occurred during the formation of HgCl_2_-tyrosinase complex. *K*_sv_ values decreased gradually with the increase of the temperatures, which manifested that the fluorescence quenching of tyrosinase by HgCl_2_ was a static quenching process (Lin et al., 2018). The corresponding *K*_q_ values of (2.14 ± 0.05) × 10^12^ and (1.83 ± 0.03) × 10^12^ L/mol/s at 298 and 304 K were much higher than the maximum scattering collision quenching constant (2.0×10^10^ L/mol/s) (Abouzied and Alshihi, 2008), suggesting that static quenching is dominant in the HgCl_2_–tyrosinase interaction (Wang et al., 2014).

For static quenching interactions, if there are similar and independent sites in biological molecules, the apparent binding constants (*K*_a_) and the number of binding sites (n) can be obtained from the following equation:

(5) $$\text{log}\frac{F_{0}-F}{F}=\text{log}K_{\text{a}}+\text{nlog}\left[\text{Q}\right]$$

According to the intercept and slope value of the regression curve (**Figure 5D**), the values of *K*_a_ and n for metal–tyrosinase interaction were calculated based on equation (5). The *K*_a_ value of (2.87 ± 0.02) × 10^4^ L/mol achieved the order of magnitude of 10^4^ L/mol, indicating the moderate binding of HgCl_2_ to tyrosinase (Fan et al., 2017). The results of the section (INHIBITION MECHANISM OF HGCL_2_ ON TYROSINASE) showed that HgCl_2_ had a significant inhibitory effect on tyrosinase that was irreversible. We suggested that HgCl_2_ might be irreversibly coordinated with the amino acid residues of tyrosinase to form the HgCl_2_-tyrosinase complexes, resulting in the conformation change of the catalytic center of tyrosinase. Furthermore, the n values (0.96 ± 0.01) were close to one, suggesting that there was a single binding site or a single class of binding sites in tyrosinase for HgCl_2_.

### Docked Conformations

**Figure 6:**
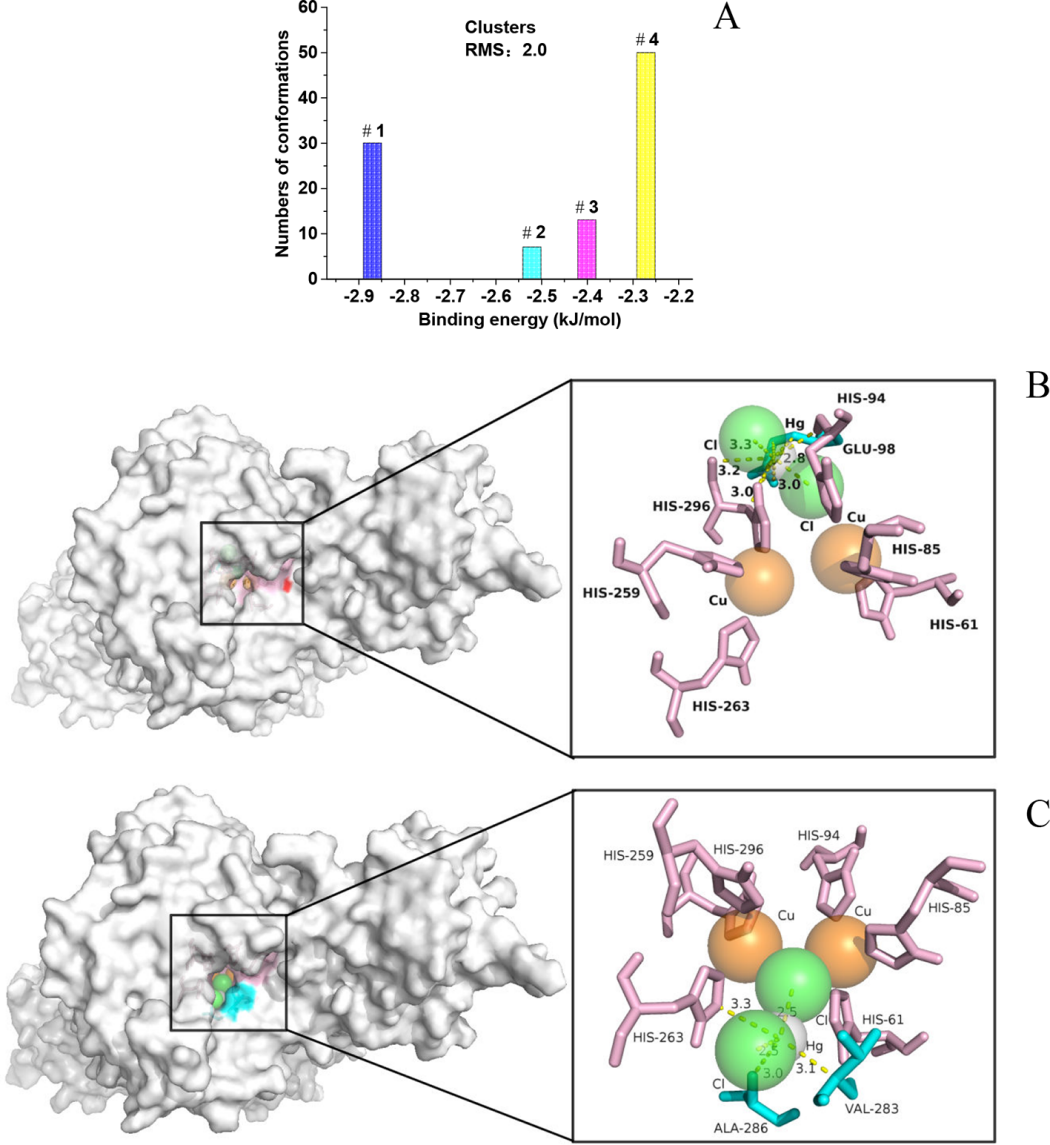
**(A)** Group the conformations into clusters at the specified RMS deviation tolerance of 2.0; **(B)** details of the optimal conformation; **(C)** details of the suboptimal conformation.

Molecular docking was furtherly performed to explore the interaction between HgCl_2_ and tyrosinase. We cluster all the conformations (100) with ADT by a root mean square (RMS) deviation tolerance of 2.0, resulting in four clusters, as shown in **Figure 6A**. The X position of each bar is plotted by the energy of the lowest energy conformation in the cluster, and the height of the bar represents the number of docked conformations in each cluster. The best docking result (optimal conformation) can be recognized as the lowest energy conformation, and can also be selected according to the RMS deviation from the reference structure (usually the crystallographic bonding mode) (Morris et al., 2008). Thus, a lowest energy (-2.87 kJ/mol) conformation in the first cluster is recognized as the optimal conformation, as shown in **Figure 6B**. A lowest energy (-2.27 kJ/mol) conformation in the fourth cluster was recognized as the suboptimal conformation because the conformation-like one with the ligand (tropolone) binds to the catalytic center of tyrosinase (Ismaya et al., 2011), as shown in **Figure 6C**. For the optimal conformation, HgCl_2_ is surrounded by amino acid residues (His 94, His 296, and Glu 98), whilst the suboptimal conformation is located adjacent to amino acid residues (His 263, Ala 286, and Val 283). The detailed information of interaction between the amino acid residues and HgCl_2_ is summarized in **Table 1**. As we all know, the catalytic center of tyrosinase is like a pocket, which consists of two Cu ions and six histidine residues, including His 61, His 85, His 94, His 259, His 263, and His 296 (Ismaya et al., 2011). Therefore, it can be inferred that the HgCl_2_ may change the morphology of catalytic center by binding with the amino acid residues, such as His 94, His 296, and His 263. Some experiments (in next section) were furtherly designed to support this point.

**Table 1:** Information between the amino acid residues and HgCl_2_.

Conformations	Binding energy (kJ/mol)	Residue	Atom of the residue^a^	Distance(Å)
Optimal	-2.87	His 94	O	2.8
		His 296	O	3.2
		His 296	CB	3.0
		His 296	HD	3.0
		Glu 98	OE	2.7
		Glu 98	ND	3.6
		Glu 98	CD	3.3
		Glu 98	CG	3.3
		Glu 98	CB	2.8
Suboptimal	-2.27	His 263	CE	3.3
		His 263	ND	3.5
		His 263	NE	3.9
		Ala 286	CB	3.0
		Val 283	CA	3.1

a The first character of the atom name consists of the chemical symbol for the atom type. All the atom names beginning with “C” are carbon atoms; “N” indicates a nitrogen and “O” indicates oxygen. The next character is the remoteness indicator code, which is transliterated according to: “B” stands for (~) “β”; “G” ~ “γ”; “D” ~ “δ”; “E” ~ “ϵ”; “Z” ~ “ζ”; and “H” ~ “η”.

### Further Exploration of Binding Sites

**Figure 7:**
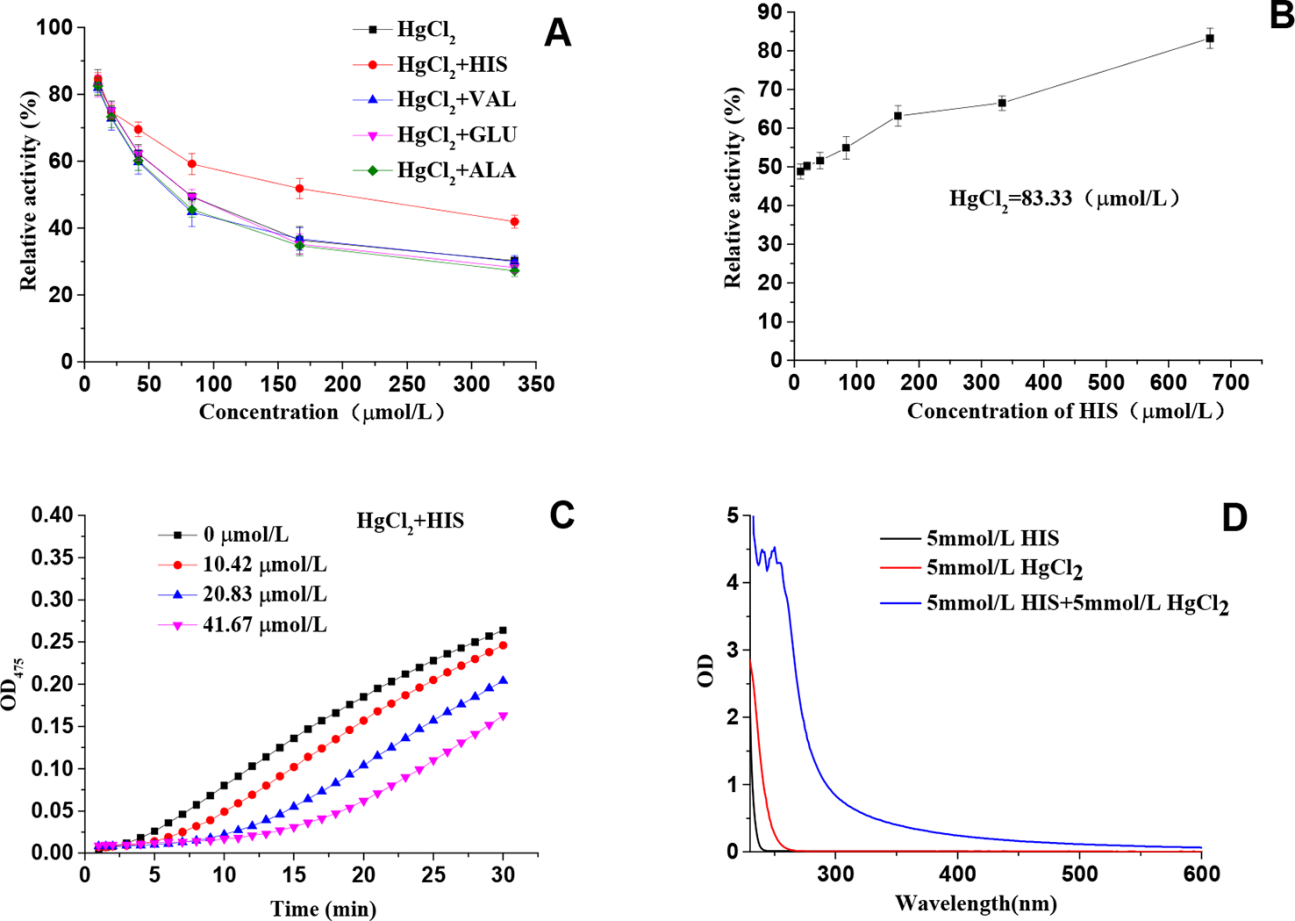
**(A)** Effects of amino acids on protecting the diphenolase activity in the presence of HgCl_2_; **(B)** The relationship between the protecting effects and the concentration of His; **(C)** Effects of His on protecting the monophenolase activity in the presence of HgCl_2_; **(D)** The UV-Vis spectra of His, HgCl_2,_ and a mixture of both. The experiment was repeated three times (n = 3).

Amino acids, including His, Val, Glu, and Ala, were checked to see whether they have any impact on the inhibitory effect of HgCl_2_ on tyrosinase. It was found that only His exhibited the significant effect on protecting the diphenolase activity in the presence of HgCl_2_, as shown in **Figure 7A**. We also found that His took effects in a concentration-dependent manner, and the protecting capability increased from 48.83 ± 1.89% to 83.43 ± 2.56% with the increasing concentrations ranged from 10.42 to 666.67 μmol/L, which is illustrated in **Figure 7B**. The effects of His on monophenolase activity in the presence of HgCl_2_ were shown in **Figure 7C**, which demonstrated that His also protect the monophenolase activity when compared to **Figure 1B**. Val, Glu, and Ala still exhibited no effects on protecting the monophenolase activity (Data no shown). The results demonstrated that HgCl_2_ preferred to bind with His rather than other amino acid residues, such as Val, Glu, and Ala. Once HgCl_2_ bound to free His, it was difficult to bind it to the residual His, which resulted in the protective effects. In order to further prove the interaction between His and HgCl_2_, the UV-Vis spectra of His, HgCl_2_, and the mixture of both is presented in **Figure 7D**; this shows the significant difference between the mixture and His (or HgCl_2_), suggesting the formation of His-HgCl_2_ complexes. Thus, based on this indirect and direct evidence (results of docking study), we believe that HgCl_2_ exhibits inhibitory effects on tyrosinase by binding to the amino residual (His) of the catalytic center of tyrosinase.

## Conclusions

In this paper, kinetics studies, different spectroscopic measurements, and molecular docking studies were employed to explore the inhibitory effects and mechanism of HgCl_2_ on tyrosinase. For monophenolase, HgCl_2_ could obviously prolong its lag time and decrease its steady-state rate with the IC_50_ value of 29.97 μmol/L. A kinetic study showed that HgCl_2_ could also significantly inhibit the diphenolase activity with the IC_50_ value of 77.93 μmol/L in an irreversible non-competitive manner. HgCl_2_ quenched the fluorescence of tyrosinase by a static procedure and formed an irreversible complex with *K*_i_ value of 29.41 μmol/L. The molecular docking study suggested that HgCl_2_ bound to the His residual of the catalytic center of tyrosinase and might change the morphology, leading to the inhibitory effects. Our studies firstly demonstrated that HgCl_2_ has the capability to inhibit tyrosinase directly, which is helpful to further our understanding of the inhibition mechanism of mercury on tyrosinase and to expand the scientific understanding of the toxicity of mercury in organisms.

## Data Availability Statement

The datasets generated for this study are available on request to the corresponding author.

## Author Contributions

JC, ZR, and NJ contributed conception and design of the study and performed the docking study. YY, MR, and QL designed and performed the inhibitory effects of HgCl_2_ on tyrosinase. All authors contributed to manuscript revisions and read and approved the submitted version.

## Funding

This work was supported by the Natural Science Foundation of Fujian Province (grant number 2019J01806), the Education Department of Fujian Province (grant number JZ160470), the Putian Science and Technology Bureau (grant number 2018SP3004), and the Training Program of Innovation and Entrepreneurship for Undergraduates (grant number 201811498003, 201811498027, and 201911498055).

## Conflict of Interest

The authors declare that the research was conducted in the absence of any commercial or financial relationships that could be construed as a potential conflict of interest.
